# Pork Loin Chop Quality and Muscle Fiber Characteristics as Affected by the Direction of Cut

**DOI:** 10.3390/foods10010043

**Published:** 2020-12-26

**Authors:** Sumin Song, Chi-Hoon Ahn, Mingeun Song, Gap-Don Kim

**Affiliations:** 1 Graduate School of International Agricultural Technology, Seoul National University, Pyeongchang 25354, Korea; suminsong@snu.ac.kr (S.S.); chihoonahn@snu.ac.kr (C.-H.A.); 2 Institutes of Green Bio Science and Technology, Seoul National University, Pyeongchang 25354, Korea; smg0508@snu.ac.kr

**Keywords:** pork loin chop, cut direction, muscle fiber characteristics, meat quality

## Abstract

In this study, the relationship between muscle fiber characteristics and meat quality of pork loin chops prepared using different directions of cut (vertical to the muscle length, M-Vertical; vertical or parallel to the muscle fiber orientation, F-Vertical or F-Parallel) was evaluated under different storage conditions (fresh, cold storage/aged, and freeze–thawed). Among the three groups, F-parallel displayed considerably larger size of muscle fibers, regardless of their type. This group also displayed an increase in discoloration in aged chops and a decrease in purge loss and tenderness than in other cut groups (*p* < 0.05). Freeze–thawing accelerated deterioration of meat quality, especially water-holding capacity and tenderness in all groups (*p* < 0.05), but was most prominent in F-Parallel. Therefore, to avoid excessive deterioration of fresh, aged, or frozen/thawed pork loin chops, it is important to consider the direction in which the chop is cut with respect to the muscle fiber orientation.

## 1. Introduction

Each skeletal muscle has unique morphological and histochemical characteristics based on its own physiological properties [1,2,3]. The skeletal muscles are divided into various muscle fiber architecture, such as parallel, fusiform, and pennate [1,4]. The pennation type (unipennate, bipennate, and multipennate) is characterized by the relative orientation and the angle formed by the skeletal muscle fibers to the force-generating axis [5,6]. The *longissimus dorsi* (LD) muscle, a large muscle on the mammalian back that is responsible for the contraction and support of the vertebrae, is a unipennate type [7]. Pennation angles of LD muscles range from 48.00° to 83.33° and from 31.36° to 53.90° for pig and cattle, respectively [8,9].

Pork and beef loin steaks for cooking are generally prepared by cutting vertical to the muscle length. Muscle fibers exposed on the surface of loin steak are elliptical in shape due to the unipennate orientation of the muscle fiber to the fascia of the loin muscle. Additionally, cross-sectional area of muscle fiber on the chop surface varies because of different angle of fiber along the length of the individual loin muscles [8]. A previous study has demonstrated that meat quality, including cooking loss and tenderness, depends on the steak position in the beef strip loin that affect the muscle fiber angle [9]. In pork loin, the shape of muscle fiber on chop surface is determined by the cut axis and the pennation angle [8].

It is known that regardless of the species, the quality of meat is closely related to the characteristics of the muscle fibers. The relative composition, size, and density of muscle fiber type IIB are established to be negatively correlated with meat color, water-holding capacity, ultimate pH, and tenderness of the meat, whereas those of oxidative fiber types I or IIA are positively correlated with water-holding capacity, tenderness, and intramuscular fat content in pork or beef [10,11,12,13,14,15]. In addition, the quality of aged pork, especially meat color, is closely related to the muscle fiber size of type IIB or the relative composition of type I/IIA fibers [16]. Although numerous studies have been widely conducted on the relationship between muscle fiber characteristics and meat quality, the influence of muscle fiber characteristics on the cut surface on the loin chop quality remains unclear.

The muscle fiber shape and size on the surface of pork loin chops are expected to differ when the meat is cut in different directions. Thus, we hypothesized that the direction of the cut also affects meat quality. To verify this hypothesis, pork loin chops were prepared by cutting in three different directions (vertical to the muscle length, and vertical and parallel to the muscle fiber orientation). For a more detailed evaluation, aged, freeze–thawed, as well as fresh pork loin chops were investigated for their meat quality in the present study.

## 2. Materials and Methods

### 2.1. Sample Preparation

**Figure 1:**
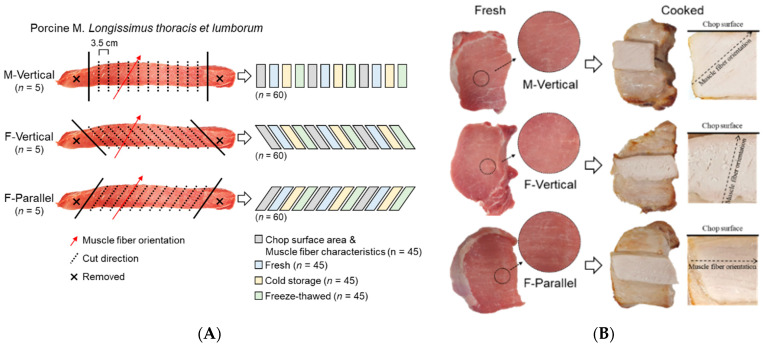
Schematic representation of pork loin (M. *longissimus thoracis et lumborum*) chop preparation. (**A**) Preparation method of pork loin chops cut in different directions: M-Vertical, cut vertical to the longitudinal direction of loin muscle; F-Vertical, cut vertical to the muscle fiber orientation; F-Parallel, cut parallel to the muscle fiber orientation. Fresh, at 1 day postmortem stored at 1 °C; Cold storage, stored at 1 °C for 7 days; freeze–thawed, frozen at −20 °C for 5 days and thawed at 1 °C for 2 days. (**B**) Representative images of fresh and cooked pork loin chops cut in different directions.

Fifteen pork loins (M. *longissimus thoracis et lumborum*; LTL) were obtained from the left side of pig carcasses (84.3 ± 3.6 kg of carcass weight; castrated; 6 months of age) at 1 day postmortem at a commercial slaughterhouse. The pigs (Landrace × Yorkshire × Duroc) were all bred on the same farm under the same breeding conditions. After slaughter, the carcasses were cooled in a cold room at 1 °C for 24 h. Pork loin was removed from cooled pig carcasses. The sample preparation is schematized in Figure 1A. The LTL muscles were randomly categorized into 3 groups (5 loins per group) and chops (3.5-cm thickness) were prepared by cutting the LTL muscles in different directions: vertical to the longitudinal axis of the loin (M-Vertical); vertical to the orientation of muscle fiber (F-Vertical) and; parallel to the muscle fiber orientation (F-Parallel). Twelve chops were cut from the medial region of the individual LTL and the remainder from both sides was removed. From each loin, three chops were selected from anterior, medial, and posterior regions for evaluating cut surface area and muscle fiber characteristics. Surface area was measured by tracing the circumference of the LTL chop on acetate paper. Three pieces of muscle cubes (1.0 × 1.0 × 1.5 cm^3^) were cut from these loin chops, frozen in chilled 2-methylbutane, and kept at −80 °C until immunohistochemical analysis. The representative images of fresh and cooked pork loin chop are shown in Figure 1B. The remaining 9 of the 12 chops were weighed individually and then vacuum-packed in plastic bags. A total of 45 chops per group were randomly subjected to three treatments, fresh (at 1 day postmortem), cold storage (kept at 1 °C for 7 days), and freeze–thawed (frozen at −20 °C for 5 days and thawed at 1 °C for 2 days).

### 2.2. pH and Meat Color

Samples (3 g) collected at 1 day postmortem (fresh group) and at 7 days of storage (cold storage and freeze–thawed groups) were homogenized with 27 mL of deionized water and the pH of homogenates was measured using a pH-meter (S220, Mettler Toledo, Greifensee, Switzerland). Prior to meat color evaluation, the pork loin chop was exposed to air for 30 min for myoglobin oxygenation. The surface color of the LTL chop was measured using a colorimeter (CR-400; Minolta Co., Tokyo, Japan) with a D65 light source after calibration using a white standard plate (Y = 93.5, x = 0.3132, y = 0.3198). The color values are presented, as defined by the Commission Internationale de l’Eclairage (CIE) [17], in terms of lightness (L*), redness (a*), and yellowness (b*).

### 2.3. Water-Holding Capacity (WHC) and Warner–Bratzler Shear Force (WBSF)

For evaluation of WHC, purge loss, drip loss, and cooking loss were measured. Purge loss was calculated by recording the sample weights before and after aging or freeze–thawing and presented as a percentage of the initial weight of the sample. Drip loss was measured using a modified version of the Honikel [18] method. Briefly, the sample (approximately 30 g) was suspended in a plastic bag at 4 °C. After 24 h, the sample was removed from the plastic bag and weighed. Drip loss was presented as a percentage of the initial weight. For cooking loss measurement, the LTL chops were cooked in a convection oven (COS-050ER, Kostem, Korea) until the internal temperature of sample reached 70 °C. After cooling at room temperature, the chop was weighed and the change in weight before and after cooking was expressed as cooking loss (%). Three cores (1.3 cm diameter) were prepared by cutting parallel to the chop surface from the posterior, medial, and anterior portions of each cooked chop after measuring cooking loss. Each core was then sheared with a V-shape blade and WBSF values were determined using a texture analyzer (TA1, Ametek, Largo, FL, USA) with adjusting fitted for WBSF. WBSF value recorded for each chop from a group was the average of the three cores.

### 2.4. Immunohistochemistry

**Figure 2:**
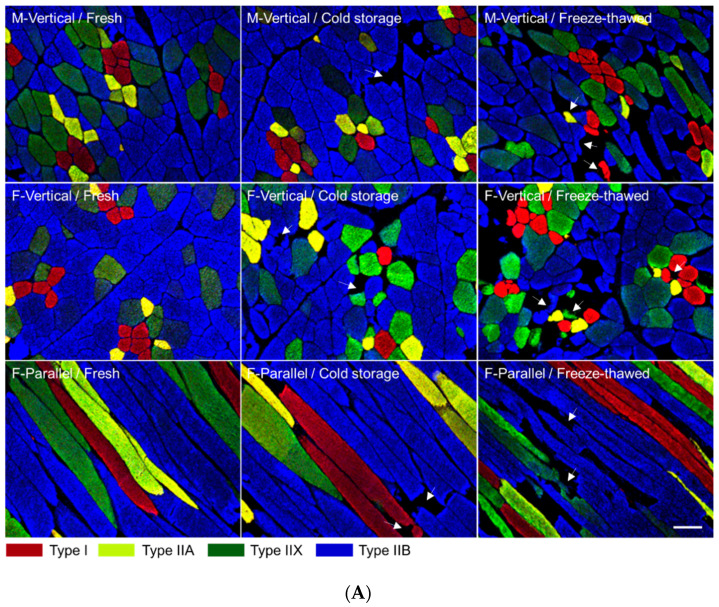

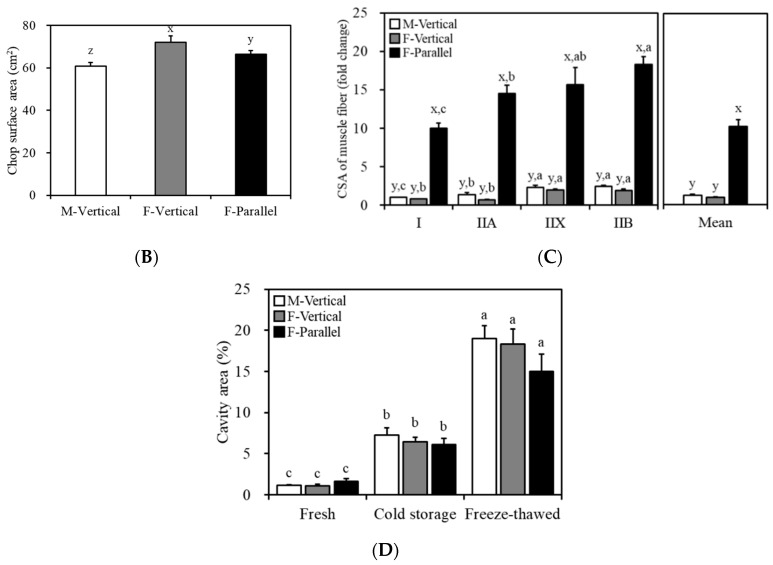
Immunohistochemical analysis and evaluation of chop surface area and muscle fiber characteristics. (**A**) Representative stained sections of pork loin chops cut in different directions: M-Vertical, cut vertical to the longitudinal direction of loin muscle; F-Vertical, cut vertical to the muscle fiber orientation; F-Parallel, cut parallel to the muscle fiber orientation. The merged images were obtained from three different sections incubated individually with antibodies, BF-35, SC-71, and BF-F3, specific for the four types (I, IIA, IIX, and IIB) of muscle fibers. Arrowheads indicate damaged muscle fibers. Bar indicates 100 μm. Evaluation of (**B**) surface area of pork loin chop, (**C**) cross-sectional area (CSA) of muscle fiber, and (**D**) cavity area. Fresh, at 1 day postmortem stored at 1 °C; Cold storage, stored at 1 °C for 7 days; freeze–thawed, frozen at −20 °C for 5 days and thawed at 1 °C for 2 days. x–z letters on the bar indicate significant difference (*p* < 0.05) between pork cut groups. a–c letters on the bar indicate significant difference (*p* < 0.05) between muscle fiber types or storage methods within the same cut group.

For staining and classification of muscle fiber types, the Song et al. [19] method, with some modifications, was used. Briefly, transverse sections (10-µm thickness) were obtained from muscle cubes using a cryostat microtome (CM1520, Leica Biosystems, Wetzlar, Germany). The sections were blocked with 10% normal goat serum (Cell Signaling Technology, Danvers, MA, USA). Three monoclonal antibodies (BF-35, SC-71, and BF-F3; DSHB, Iowa City, IA, USA) specific to myosin heavy chain (MHC) isoforms, MHCs I/slow and 2a, MHCs 2a and 2x, and MHC 2b, respectively, were incubated with a section. Fluorescent dye-conjugated anti-mouse IgG (Alexa Fluor 488 and 594; Thermo Fisher Scientific, Waltham, MA, USA) or anti-mouse IgM (Alexa Fluor 405; Thermo Fisher Scientific) were used as secondary antibodies. All slides were visualized by a confocal scanning laser microscope (TCS SP8 STED, Leica Biosystems, Wetzlar, Germany). Muscle fibers were identified and classified into the four types (I, IIA, IIX, and IIB) based on the distribution of MHCs detected by the primary antibodies against MHCs I, 2a, 2x and 2b, respectively (Figure 2A). Approximately 600 fibers of each section were analyzed for determining muscle fiber characteristics, such as cross-sectional area (CSA; μm^2^), relative fiber number (%), relative fiber area (%), and cavity area (%) using an Image Pro Plus Program (Media Cybernetics, Rockville, MD, USA).

### 2.5. Statistical Analysis

Experimental data are presented as means with standard error. For statistical analysis, the SAS 9.4 software (SAS Institute, Cary, NC, USA) was used. The effects of the direction of cut and storage method (fresh, cold storage, and freeze–thawed) on pork chop quality (pH, meat color, WHC, and WBSF), chop surface area, and muscle fiber characteristics (CSA, cavity area, relative fiber number, and relative fiber area) were evaluated using the PROC ANOVA procedure and Duncan’s multiple range test of the SAS 9.4 software. Statistical significance was accepted at *p* < 0.05.

## 3. Results

### 3.1. Surface Area and Muscle Fiber Characteristics of Pork Loin Chops

The muscle fibers on the surface of loin chops cut in different directions were evidently distinct in shape (Figure 2A). The chop surface area was the largest in the F-Vertical group and the smallest in the M-Vertical group of the pork loin chops (*p* < 0.05; Figure 2B). In F-Parallel, the muscle fibers exposed on the surface were longitudinal in shape and therefore, presented the largest CSA regardless of the type of muscle fiber (*p* < 0.05; Figure 2C). However, no significant difference between the M-Vertical and F-Vertical groups was observed in the CSA of all types of muscle fibers, which were more or less ellipse-shaped. In both these groups, the CSA of types IIB and IIX was larger than that in type I (*p* < 0.05). Interestingly, the CSA observed for type IIA was smaller than those in types IIX and IIB in both M-Vertical and F-Vertical groups (*p* < 0.05), but was not different from that in type IIX in F-Parallel (*p* > 0.05). Type I showed the lowest CSA among the fiber types in M-Vertical and F-Parallel (*p* < 0.05), but was not significantly different from other fiber types in F-Vertical. The cavity area (%) was not influenced by the direction of the cut (*p* > 0.05), but was affected by whether the pork chops were fresh, aged in cold storage, or freeze–thawed (Figure 2D). The highest cavity area was observed in freeze–thawed chops (*p* < 0.05) and their percentages ranged from 15.01% to 18.98%. The cavity area percentage was the lowest in fresh chops regardless of the direction of the cut (1.08%–1.63%; *p* < 0.05). The cavity area percentage of aged chops showed higher values compared to those in the fresh ones but lower values than those in the freeze–thawed chops (*p* < 0.05).

Type IIB was found to dominate more than half the muscle fiber composition in M-Vertical and F-Vertical (*p* < 0.05; Table 1). In F-Parallel, both type IIB and IIX were the predominant muscle fibers with nearly equal prevalence (*p* > 0.05). Type IIA was the least dominant muscle fiber type in all groups (*p* < 0.05). The relative number of type I muscle fibers was higher than that of type IIA but lower than that of type IIX and IIB in all groups (*p* < 0.05). A similar trend was observed for the relative area, though in F-parallel, type IIB covered a higher area than type IIX (*p* < 0.05). F-Parallel had higher proportions (relative number and area) of type I, IIA, and IIX than the other two groups (*p* < 0.05), whereas type proportions of IIB were lower in F-Parallel than those in M- or F-Vertical (*p* < 0.05). The F-Vertical group had the highest proportions of type IIB but the lowest compositions of type IIA (*p* < 0.05).

### 3.2. Comparison of Meat Quality of Pork Loin Chops Cut in Different Directions

The results of pH and meat color of pork loin chops cut in different directions and kept under various storage conditions are presented in Table 2. No significant differences were observed in the pH within the fresh and freeze–thawed groups of pork loin chops cut in different directions. However, the pH of pork loin chop aged in cold storage showed a significant drop in value and was lowest in M-Vertical followed by F-Vertical and F-Parallel. When compared to fresh chops, the pH values of all other chops the increased (*p* < 0.05), except for the cold storage-M-Vertical chops. In fresh pork loin chops, the lightness (L*) and redness (a*) values were lowest in F-Vertical (*p* < 0.05), while yellowness (b*) was not significantly different among the groups. Under the same storage conditions, the meat chops cut in different directions did not show any significant changes in their color traits. When compared to fresh pork chops, the ones aged in cold storage showed an increase in the values of all color traits in the M-Vertical and F-Vertical groups (*p* < 0.05), whereas the F-Parallel group showed an increase only in yellowness (*p* < 0.05). Lightness of pork loin chops was not influenced by the freeze–thawing treatment regardless of the direction of the cut (*p* > 0.05). Freeze–thawing of pork loin chops led to a decrease in redness and an increase in yellowness in the M-Vertical and F-Vertical groups, respectively (*p* < 0.05).

**Figure 3:**
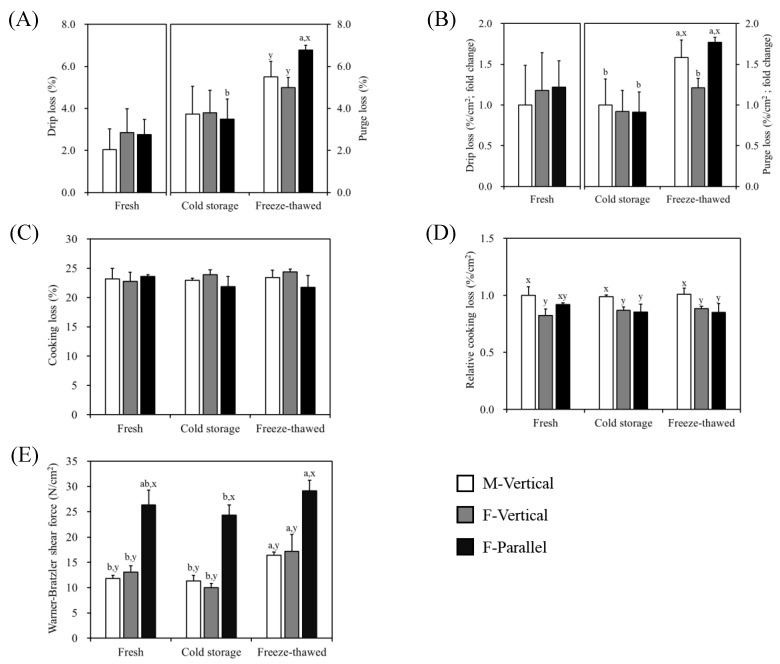
Comparison of water-holding capacity and tenderness of pork loin chops cut in different directions. (**A**) Drip loss of fresh chop and purge loss of aged or freeze–thawed chops. (**B**) Relative drip or purge losses per unit area of chop surface. (**C**) Cooking losses of fresh, aged, and freeze–thawed pork loin chops. (**D**) Relative cooking loss per unit area of chop surface. (**E**) Warner-Bratzler shear force of fresh, aged, and freeze–thawed pork loin chops. M-Vertical, cut vertical to the longitudinal direction of loin muscle; F-Vertical, cut vertical to the muscle fiber orientation; F-Parallel, cut parallel to the muscle fiber orientation. Different letters on the bar indicate significant differences between loin cut groups (x, y) or between storage methods (a, b) at *p* < 0.05.

The amount of meat exudates (drip loss or purge loss) did not show any significant difference in fresh and aged pork loin chops of all groups (*p* > 0.05), as shown in Figure 3A. However, purge loss in freeze–thawed loin chops was affected by the direction of the cut as the F-Parallel group had higher purge loss than other groups (*p* < 0.05). Further, though all freeze–thawed chops displayed a greater purge loss than that exhibited by the aged chops, it was more only in the F-Parallel group (*p* < 0.05). The values of relative drip loss or purge loss per unit area (%/cm^2^) within the fresh or aged groups of pork loin chops were not significantly different; these values of freeze–thawed M-Vertical and F-Parallel groups were higher than those of freeze–thawed F-Vertical (*p* < 0.05; Figure 3B). Additionally, higher values were observed in M-Vertical and F-Parallel groups subjected to freeze–thawing than when aged in cold storage (*p* < 0.05). The cooking loss was not significantly different between the pork loin chops, regardless of the direction of the cut and storage methods (Figure 3C). However, relative cooking loss per unit area of chop surface showed the highest ratio in M-Vertical group than in other cut groups regardless of storage methods (*p* < 0.05; Figure 3D). Both F-Vertical and F-Parallel groups showed similar relative cooking loss (*p* > 0.05). Additionally, storage methods did not affect the relative cooking loss within the same cut group (*p* > 0.05).

WBSF values of pork loin chops were higher (*p* < 0.05) in F-Parallel group than in other cut groups regardless of storage methods. No differences (*p* > 0.05) were noted between M-Vertical and F-Vertical groups. Within the same cut group, an increase in WBSF value due to freeze–thawing was observed in the M-Vertical and F-Vertical groups (*p* < 0.05), whereas that of F-Parallel group was neither affected by freeze–thawing nor cold storage treatment (*p* > 0.05; Figure 3E). A slight decrease in WBSF due to cold storage was found in the M-Vertical and F-Vertical, but not significant.

## 4. Discussion

The chop surface characteristics depend on the surface area (loin eye) and the degree of angle in the direction of the muscle fibers [1,8]. The unipennate muscle fibers on the surface of pork loin chops varied in shape and size depending on the direction of cut, as illustrated in Figure 2A,C. Relatively larger muscle fibers were observed on the surface of the F-Parallel group, regardless of muscle fiber types owing to the elongated cylindrical shape of the muscle fibers [20,21]. Among the muscle fiber types, IIB or IIX fibers are generally observed to be larger than types I or IIA, regardless of species and breeds [19,22,23]. Our results, regardless of cut groups, corroborated with the previous observations. Additionally, the muscle fiber compositions in M-Vertical and F-Vertical groups were found to be similar to those reported by previous studies that demonstrated that glycolytic fiber (IIX or IIB) content was higher than the oxidative fiber (I or IIA) content in pork loin [19,24,25,26]. Notably, the F-Parallel group exhibited a nearly double type I and IIA fiber content and nearly half of type IIB fiber content when compared to the M-Vertical and F-Vertical groups. The muscle fiber length as well as CSA is dependent on muscle fiber type and results in different ratios of CSA and length of muscle fiber [8,27,28]. Accordingly, muscle fibers on chop surface of F-Parallel group showed relatively higher content of type I or IIA.

The meat quality assessment of the pork loin chops revealed that only surface color and tenderness of fresh and aged chops were significantly affected by the direction of the cut, whereas all traits of meat quality of freeze–thawed pork chops were influenced by the direction of cut. Reduced tenderness in the F-Parallel group regardless of storage methods may be the result of presence of larger and elongated muscle fibers on chop surface as compared to the smaller and oblique muscle fibers of the M-Vertical or F-Vertical groups. This observation is supported by a previous study that demonstrates that larger size of muscle fiber directly correlates to the toughness of meat [29,30,31]. Aging of the meat under cold storage conditions affected the pH and meat color in a cut method-dependent manner. The increase in pH values in the F-Vertical and F-Parallel groups was consistent with previous reports that demonstrated increase in pH during aging process might be due to release of nitrogenous compounds from proteins by proteolysis [32]. The values of the meat color traits tend to increase with age due to myoglobin oxidation [33,34,35]. However, we did not see any significant changes in the lightness and redness of the F-Parallel group chops. It was expected that the tenderness would improve due to proteolysis during cold storage [36], but the WBSF did not significantly decreased after 7 days of cold storage. This is due to 3.49%–3.80% of meat exudates excreted from meat during storage. As previously reported [37], meat with better water-holding capacity or water distribution is tenderer than meat with less water-holding capacity.

Contrary to the observations in aged pork loin chops, while freeze–thawing dramatically accelerated the deterioration of the meat quality including tenderness, water-holding capacity, and pH of pork loin chops regardless of the direction of the cut, it did not affect the meat color. For the water-holding capacity attributes, cooking loss was not affected by freeze–thawing regardless of the direction of cut, despite increased purge loss and drip loss. This is because large amounts of water are exuded, resulting in less water that can be released from the freeze–thawed chop. This result is supported by previous research showing that cooking loss is affected by the water fractions, which can be changed by freeze–thawing and aging of meat [38,39]. Freeze–thawing leads to an increase in hydrogen ion concentration within muscles due to loss of water resulting in a decreased pH [40]. However, our results (increased pH) was contrary to a previous report. Increased pH in this study seems to be due to the proteolytic release of free amino acids and dipeptides in the sarcoplasm as reported earlier [41,42]. The direction of cut did not affect pH of freeze–thawed pork loin chop. The water-holding capacity (especially purge loss) and tenderness highly deteriorated in F-Parallel than in other cut groups. Previously, we demonstrated that muscle fiber type II was more susceptible to the detrimental effects of freezing than type I fibers in terms of water-holding capacity and tenderness of meat [43]. Additionally, muscles having higher content of oxidative fibers (I and IIA) are more stable after freezing than the muscles composed of large amount of glycolytic fibers [44]. Contrary to these findings, our results show a higher content of type I in muscle fiber composition on chop surface in F-Parallel than in the others. Moreover, the cavity areas formed due to ice crystals were similar among the cut groups (Figure 2D). Therefore, we propose that deterioration of meat quality of freeze–thawed pork chops cut parallel to the muscle fiber orientation is due to the longitudinal (as opposed to transverse or oblique in other cut groups) morphology of the muscle fibers rather than their composition on the surface of pork chop. It is known that the meat discoloration is accelerated by freeze–thawing due to myoglobin degradation, reduction of reductase activity, and lipid oxidation [45,46]. Freeze–thawing also affects the ratio of different chemical state of myoglobin, i.e., met-, oxy-, and deoxy-myoglobin; however, most of these studies have assessed beef that has a higher content of myoglobin than that in pork [47,48,49]. Our meat color results also demonstrate that pork, which has lower myoglobin content than beef, is not much affected by freeze–thawing.

## 5. Conclusions

The direction of the cut did not significantly affect the quality of fresh pork chop except for tenderness, but it resulted in the discoloration of aged pork chops and the deterioration of water-holding capacity and tenderness of freeze–thawed pork chops. Especially, the F-parallel group (cut parallel to the muscle fiber orientation) exhibited the poorest meat quality, regardless of storage methods. In conclusion, to avoid deterioration of pork chop, the direction of the cut with respect to the muscle fiber orientation needs to be considered when preparing pork loin chops, especially when stored under frozen conditions.

## Figures and Tables

**Table 1:** Comparison of muscle fiber composition between pork loin chops cut in different directions.

Traits	Muscle Fiber Type	Direction of Cut ^(1)^					
		M-Vertical		F-Vertical		F-Parallel	
Relative fiber number (%)	I	12.25	±1.09 ^c,y^	11.96	±1.32 ^c,y^	20.10	±1.82 ^b,x^
	IIA	8.88	±1.52 ^d,z^	5.34	±1.64 ^d,y^	12.40	±1.51 ^c,x^
	IIX	20.56	±1.17 ^b,y^	16.76	±1.68 ^b,z^	32.79	±1.78 ^a,x^
	IIB	58.31	±2.24 ^a,x^	65.94	±2.05 ^a,x^	34.70	±3.05 ^a,z^
Relative fiber area (%)	I	6.66	±0.76 ^c,y^	6.67	±1.12 ^c,y^	15.35	±1.38 ^c,x^
	IIA	5.62	±1.15 ^c,y^	2.56	±0.65 ^d,z^	12.60	±1.56 ^d,x^
	IIX	22.00	±3.64 ^b,y^	18.69	±1.26 ^b,y^	33.12	±1.24 ^b,x^
	IIB	65.72	±3.84 ^a,y^	72.08	±2.15 ^a,x^	38.93	±1.45 ^a,z^

^a–d^ Means ± SE with different superscripts are significantly (*p* < 0.05) different within the same column. ^x–z^ Means ± SE with different superscripts are significantly (*p* < 0.05) different within the same row. ^(1)^ M-Vertical, cut vertical to the longitudinal direction of loin muscle; F-Vertical, cut vertical to the muscle fiber orientation; F-Parallel, cut parallel to the muscle fiber orientation.

**Table 2:** Comparison of pH and meat color of pork loin chops cut in different directions.

Meat Color	Direction of Cut ^(1)^	Storage Methods ^(2)^					
		Fresh		Cold Storage		Freeze–Thawed	
pH	M-Vertical	5.46	±0.02 ^y^	5.46	±0.01 ^b,y^	5.59	±0.05 ^x^
	F-Vertical	5.43	±0.04 ^z^	5.68	±0.03 ^a,x^	5.58	±0.02 ^y^
	F-Parallel	5.44	±0.04 ^z^	5.61	±0.05 ^a,x^	5.55	±0.06 ^y^
CIE L*	M-Vertical	50.37	±2.21 ^ab,y^	53.64	±2.58 ^x^	48.48	±2.16 ^y^
	F-Vertical	49.78	±2.22 ^b,y^	54.56	±3.38 ^x^	51.73	±2.81 ^x,y^
	F-Parallel	52.72	±2.29 ^a^	53.00	±1.91	50.06	±2.64
CIE a*	M-Vertical	6.26	±1.65 ^a,x^	6.47	±0.92 ^x^	5.78	±1.30 ^y^
	F-Vertical	4.86	±0.79 ^b,y^	6.95	±2.07 ^x^	6.37	±1.40 ^xy^
	F- Parallel	6.43	±1.34 ^a^	6.79	±0.87	5.66	±1.23
CIE b*	M-Vertical	4.87	±1.17 ^y^	8.01	±1.38 ^x^	6.89	±1.46 ^x,y^
	F-Vertical	4.53	±1.01 ^y^	8.50	±1.54 ^x^	8.20	±1.82 ^x^
	F- Parallel	5.61	±0.92 ^y^	7.99	±0.62 ^x^	6.83	±2.00 ^x,y^

^a,b^ Means ± SE with different superscripts are significantly (*p* < 0.05) different within the same column. ^x–z^ Means ± SE with different superscripts are significantly (*p* < 0.05) different within the same row. ^(1)^ M-Vertical, cut vertical to the longitudinal direction of loin muscle; F-Vertical, cut vertical to the muscle fiber orientation; F-Parallel, cut parallel to the muscle fiber orientation. ^(2)^ Fresh, at 1 day postmortem stored at 1 °C; cold storage, stored at 1 °C for 7 days; freeze–thawed, frozen at −20 °C for 5 days and thawed at 1 °C for 2 days.

## Data Availability

Data sharing not applicable.
